# miR-17: from developmental regulatory hub to molecular engine driving tumourigenesis

**DOI:** 10.3389/fonc.2026.1787691

**Published:** 2026-05-20

**Authors:** Min Zhang, Yurong Ma, XuanYu Chen, Tiansi Sun, XiYi Wang, Feng Hui Wang

**Affiliations:** Yan’an Medical College of Yan’an University, Yan’an, Shaanxi, China

**Keywords:** diagnostic markers, miR-17, signalling pathways, target genes, tumor biology

## Abstract

MicroRNA-17(miR-17) is a prototypical oncogenic miRNA that plays a central driving role in the initiation and progression of various malignant tumours (MT), including lymphoma, lung cancer, colorectal cancer (CRC), and breast cancer (BC). It inhibits the activation of apoptosis pathways and promotes the G1/S phase transition by targeting tumor suppressor genes and cell cycle regulators, thereby driving the unlimited proliferation of tumor cells. Concurrently, it targets molecules associated with epithelial-mesenchymal transition (EMT), activating key signalling pathways, such as Phosphatidylinositol 3-Kinase/Protein Kinase B (PI3K/AKT) and Wnt/Beta-catenin(Wnt/β-catenin) to enhance the invasiveness and migration capabilities of tumour cells. Moreover, miR-17 participates in a cross-regulatory network of non- coding RNAs(ncRNAs), acting both as a molecular sponge to sequester long non-coding RNAs(lncRNAs) and circular RNAs(circRNAs), and as a regulator of the stability or biogenesis of these ncRNAs.This further amplifies oncogenic effects, induces tumor cell resistance to chemotherapy and radiotherapy, and ultimately synergistically promotes tumor angiogenesis, thereby remodeling the tumor microenvironment(TME). In clinical applications, miR-17 holds potential for early cancer diagnosis, particularly serving as a non-invasive biomarker in lung and Gastric cancers (GC). As a therapeutic target, restoring miR-17 expression via targeted interventions enhances. The efficacy of chemotherapy or targeted therapy thereby improves patient prognosis. Despite extensive research in this field, the precise mechanism of action for miR-17 remains incompletely understood. This review summarizes the current state of research concerning the relationship between miR-17 and its target genes in MT, as well as the underlying mechanisms involved.

## 1 Introduction

Despite advances in cancer prevention, early detection, and treatment, cancer remains a major unresolved medical challenge worldwide. In 2022, nearly 20 million new cancer cases were diagnosed globally, with approximately 9.7 million deaths. China recorded 4.8247 million new cases and 2.5742 million deaths, ranking first in the world for both metrics (1). The five leading causes of cancer deaths in China are lung cancer, liver cancer (LC), GC, CRC, and oesophageal cancer (EC). Among these, LC mortality rose from third place in 2018 to second place in 2022 (2). The global incidence of cancer continues to increase, placing pressure on public health systems (3). The initiation and progression of MT are primarily driven by dysregulation of gene expression, particularly within key regulatory pathways governing cell proliferation, survival, and apoptosis (4). Despite advances in understanding molecular mechanisms, the complexity and heterogeneity of cancer remain major challenges for effective treatment. However, approximately 40% of cancer deaths are associated with controllable risk factors. By managing these risks and promoting early diagnosis and precision treatment, the burden of cancer can be effectively reduced (5).

NcRNAs, including miRNAs, lncRNAs, and circRNAs, have garnered significant attention due to their pivotal roles in regulating gene expression and tumour behaviour (6). Among ncRNAs, miRNAs are the most extensively studied. They play a central role in regulating key biological processes in cancer, including cell proliferation, apoptosis, migration, and invasion (7). miRNAs constitute a class of endogenous single-stranded ncRNAs ranging from 19 to 25 nucleotides in length. Characterised by high conservation, developmental regulation, and tissue specificity, they primarily function by complementary pairing between their seed sequences and the 3’ untranslated regions (3’UTRs) of target messenger RNA (mRNA). This interaction facilitates mRNA degradation or translation inhibition, thereby regulating gene expression at the post-transcriptional level (8). miR-17 is a prototypical oncogenic miRNA that plays a pivotal role in the initiation and progression of various malignancies, including lymphoma, lung cancer, CRC, and BC. Through mechanisms such as targeting tumour suppressor genes, regulating cell cycle and signalling pathways, and participating in ncRNA cross-regulatory networks, it acts as a key driver of tumour progression (9). Among these, miR-17-5p is a core member of the miR-17–92 gene cluster, a classic oncogenic miRNA cluster identified around 2005. This cluster comprises six mature miRNAs, including miR-17-5p, miR-18a, and miR-19a (10, 11). The mature sequences of miR-17-3p and miR-17-5p are 5’-CAAAGUGCUUACAGUGCAGGUAG-3’ and 5’- UGUGCUGCUUUCUUGGGUCGG-3’ (12, 13). The human miR- 17 cluster is located at chromosome 13 long arm, region 3, band 1, subband 3(13q31.3). Its biogenesis process begins with transcription by RNA polymerase II, yielding primary miRNA(pri-miR)-17–92 containing multiple hairpin structures (14).Subsequently, within the cell nucleus, the pri-miR-17–92 transcript is cleaved by the microprocessor complex (Drosha ribonuclease III/DGCR8) into a precursor of ~70 nt, pre-miR-17 (15).Subsequently, pre- miR-17 forms a trimer with (Ras-related nuclear protein-guanosine triphosphate) Exportin-5/Ran-GTP and is transported to the cytoplasm via the nuclear pore complex. Within the cytoplasm, Dicer nucleases recognise the stem-loop structure of pre-miR-17, cleaving it to generate a double-stranded complex of approximately 22 nt comprising miR-17-5p and miR-17-3p.Ultimately, the double- stranded complex binds to the Argonaute protein(AGO protein), where miR-17-3p is degraded while miR-17-5p is retained and assembled into the RNA-induced silencing complex (RISC). This complex binds to the 3’ UTR of the target mRNA via complementary base pairing, thereby regulating the expression of the target gene (16, 17). Research has revealed that miR-17inhibits osteoblast differentiation by targeting the SRY-box transcription factor 6 (Sox6), thereby providing a potential therapeutic target for the treatment of orthopaedic conditions such as osteoporosis and osteoarthritis (18). miR-17 inhibits the progression of osteoarthritis by reducing chondrocyte apoptosis and extracellular matrix degradation through targeted suppression of Enhancer of Zeste Homolog 2( EZH2) (19). Concurrently, miR-17 alleviates pathological cardiac fibrosis by targeting BCL2/adenovirus E1B 19 kDa protein-interacting protein 3(BNIP3), thereby reducing mitochondrial autophagy in cardiomyocytes (20). Furthermore, inhibition of miR-17 alleviates acute respiratory distress syndrome (ARDS)-associated pulmonary fibrosis by modulating mitochondrial autophagy mediated by mitochondrial fusion protein 2(Mfn2) (21). More importantly, miR-17 has the potential to selectively target multiple genes, thereby inhibiting tumour growth. Its regulation of signalling pathways constitutes a key factor in tumour development, making it a promising candidate for an important therapeutic target in cancer treatment. This paper analyzes the significance of miR-17 in the field of cancer therapy, elucidating its multifaceted roles in tumor cell invasion, metastasis, proliferation, apoptosis, and drug resistance, to provide a reference for cancer treatment.

## 2 The molecular mechanisms of miR-17 in cancer

MiR-17-5P was first identified in human tumour tissue in 2005, originating from the miR-17–92 gene cluster located within an amplified region of the human chromosome 13q31 locus (22). The mechanism by which miR-17 regulates gene expression involves post-transcriptional control through basepairing between its seed sequence and the 3’- UTR of target mRNAs:miR-17 is first transcribed into pri-miRNA by RNA polymerase II within the cell nucleus. It is subsequently processed by the Drosha enzyme into pre-miRNA, before being cleaved by the Dicer enzyme into mature miRNA. Mature miRNAs are incorporated into the RISC, where they bind complementarily to the 3’-UTR of target mRNAs via their seed sequences. This leads to mRNA degradation or translational repression, thereby regulating protein expression levels and cellular processes such as proliferation, apoptosis, and metastasis (23). For example, miR-17 suppresses the proliferation of hepatocellular carcinoma(HCC) and BC cells by targeting the proto-oncogene MYC (c-Myc) (24). It may also promote tumour cell growth by suppressing the tumour suppressor genes cyclin-dependent kinase inhibitor 1A(p21) and phosphatase and tensin homolog(PTEN) (25).Furthermore, online databases such as miRNA Database(miRDB), TargetScan, and miRNA Target Basecan predict the downstream target genes of miR-17, thereby providing support for research into its functional mechanisms.

## 3 miR-17 as a molecular sponge within the competitive endogenous RNA network

CeRNA networks mediate post-transcriptional interactions between transcripts through competitive mechanisms involving shared miRNA response elements (MREs), functionally linking protein-coding mRNAs with ncRNAs.These networks encompass diverse ncRNA types, including miRNAs, lncRNAs, pseudogenes, and circRNAs.Among these, ncRNAs such as lncRNAs and circRNAs frequently act as ‘molecular sponges’ to adsorb miRNAs. By competitively binding to miRNAs, they diminish the latter’s targeting of specific mRNAs, thereby regulating the transcriptional stability or translational efficiency of target mRNAs. These mechanisms are extensively involved in crucial biological processes, including cell proliferation, migration, invasion, autophagy, and tumour drug resistance (26). In terms of cancer-promoting studies (e.g., Duan et al.) has been shown that the lncRNA LINC00957 exerts a pro-tumor effect by sequestering miR-17, thereby reducing miR-17’s inhibitory effect on its target gene natriuretic peptide (NPNT). This activation of NPNT downstream pathways (cell cycle and migration-related) ultimately promotes the malignancy of glioblastoma (27). Furthermore, lncRNA HCP5 binds to miR-17, thereby upregulating the target gene homoeobox A7 (HOXA7) and promoting proliferation, invasion, and EMT in lung adenocarcinoma cells (LAC) (28).Concurrently, the lncRNA H19 sequesters miR-17 via a competitive endogenous RNA mechanism, thereby regulating the expression of the proto-oncogene tyrosine protein kinase Yes1 (YES1) and participating in the initiation and progression of thyroid carcinoma(TC) (29). On the other hand, miR-17 exerts an anti-tumor effect. As demonstrated by Liu et al.’s research, upregulation of miR-17 reverses the inhibitory effect of lncRNA LINC01939 on GC metastasis and EMT (30). Similarly, Yan et al. (31) found that the LncRNA DNMBP-AS1 sequestered miR-17, thereby releasing its suppression of the target gene NHL repeat-containing protein 3 (NHLRC3) and consequently inhibiting the proliferation and migration of CRC cells. Gan et al. (32) discovered that by binding to miR-17, the lncRNA PTENP1 acts as a phosphoregulator and ceRNA to PTEN, thereby inhibiting the progression of bladder cancer (bladder Ca). The above studies indicate that miR-17 serves as a key node in the ceRNA network regulating tumor progression in various cancer types. Different lncRNAs competitively bind to miR-17, exerting either pro-cancer or anti-cancer effects, thereby providing potential targets for tumor-targeted therapy.

MiR-17 has also been described as an important diagnostic biomarker in cancer. For instance, Huan et al. (33) observed that in non-small cell lung cancer(NSCLC), LncRNAH19 expression was significantly elevated whilst miR-17 expression was markedly reduced, with the two exhibiting a negative correlation. Zhang et al. (34) observed that LncRNA LINC01094 expression was significantly elevated in GC tumours compared to adjacent normal tissue, and exhibited a negative correlation with miR-17expression.

MiR-17 may also indirectly contribute to chemotherapy resistance in tumour cells by forming a ceRNA network with lncRNA.For instance, Xu et al. (35) discovered that the lncRNA HCG11 sequesters miR-17 via a ceRNA mechanism, thereby reducing its inhibitory effect on the target gene p21. Ultimately, by upregulating p21 expression, LncRNA HCG11 participates in the pathological process of gefitinib resistance in NSCLC cells. Moreover, Research by Tie et al. (36) revealed that LncRNA HOTAIR M1 binds to and regulates miR-17 via a ceRNA mechanism, thereby relieving its suppression of the target gene B-cell translocation gene3 (BTG3). Ultimately, by activating the miR-17/BTG3 pathway, it participates in the process of CRC cell resistance to 5-fluorouracil (5-FU).

MiR-17 also acts as a sponge for circRNAs in MT. For instance, circMAPKBP1 activates the autophagy pathway by upregulating miR-17, thereby relieving its suppression of the target gene transforming growth factor beta 2 (TGFβ2), ultimately conferring cisplatin resistance in squamous cell carcinoma of the tongue(TSCC) (37). Concurrently, Yang et al. (38) found that circITCH modulates miR-17 through sponging, thereby upregulating its target genes p21 and PTEN. This establishes a circITCH/miR-17/p21/PTEN regulatory axis, ultimately exerting a tumour-suppressing effect in bladder Ca. Furthermore, Hong et al. (39) demonstrated that circAKT3 promotes the malignant behaviour of oesophageal carcinoma both *in vitro* and *in vivo* by sponging miR-17, thereby providing a potential therapeutic target for the treatment of EC.

In summary, miR-17 functions as a molecular sponge for lncRNAs and circRNAs, playing a pivotal role in tumourigenesis, progression, and therapeutic resistance.

**Figure 1:**
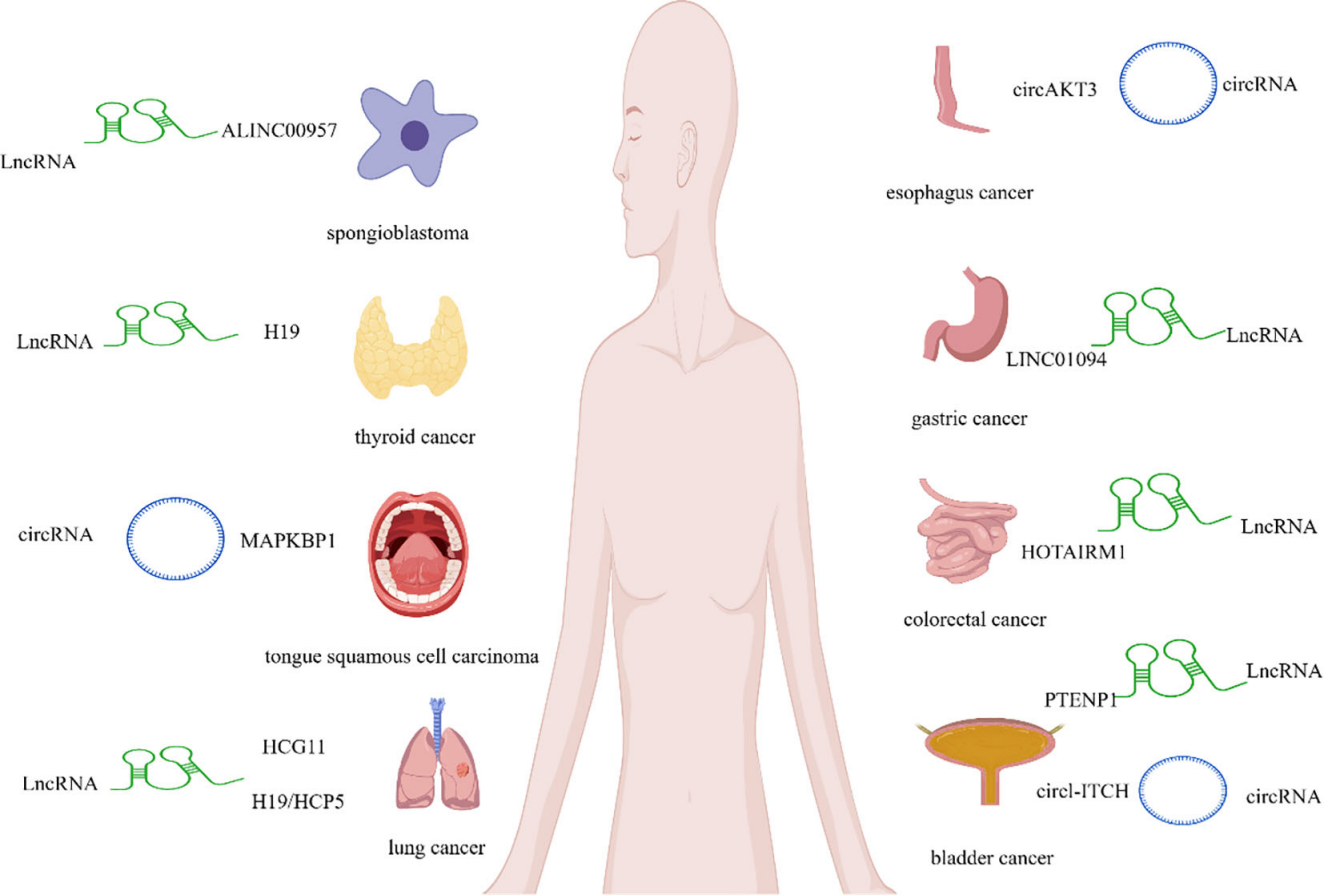
Interactions between miR-17 and lncRNA and circRNA.

Details are presented in **Table 1** and **Figure 1**.

**Table 1:** miR-17 ceRNA network and its role in cancer.

First/author/s, year	CeRNA-associated cancer	Control mechanism		(Refs.)
Duan, et al.2024	LncRNALINC00957	Glioblastoma	Upregulation of miR-17 activates cell cycle and migration pathways downstream of NPNT.	(27)
Lin, et al.2016	LncRNA H19	TC	Upregulates miR-17, modulating YES1 expression	(29)
Qing, et al.2024	lncRNA HCP5	Lung adenocarcinoma	Upregulates miR-17, enhancing expression of target gene Homology Box A7.	(28)
Zhang,eta.l2024	LncRNA LINC01094	GC	Interacts with miR-17, potentially serving as a diagnostic biomarker for cancer	(34)
Yang,et al.2022	lncRNA DNMBP-AS	CRC	Binds miR-17, releasing its inhibition on the target gene NHLRC3	(31)
Gan,etal.2017	lncRNA PTENP1	Bladder Ca	Upregulates miR-17 to inhibit PTEN	(32)
Huang,etal.2018	LncRNA H19	NSCLCr	Inversely correlated with miR-17, potentially serving as a diagnostic biomarker for cancer.	(33)
Xu,etal.2014	lncRNA HCG11	NSCLC	Regulates p21 expression by binding miR-17-5p	(35)
Tie, et al.2019	LncRNA HOTAIRM1	CRC	Colorectal cancer binds miR-17, releasing its inhibition of the target gene BTG3.	(36)
Shu,et al.2024	Circ MAPKBP1	TSCC	Upregulates miR-17, relieving its inhibition of target gene TGFβ2 and activating the autophagy pathway.	(37)
Yang,etal.2018	Circ ITCH	Bladder Ca	Upscales its target genes, p21 and PTEN, by sponging miR-17	(38)
Hang, et al.2021	CircAKT3	EC	Upscales its target genes p21 and PTEN by sponging miR-17	(39)

## 4 The role of miR-17 in cancer progression

**Figure 2:**
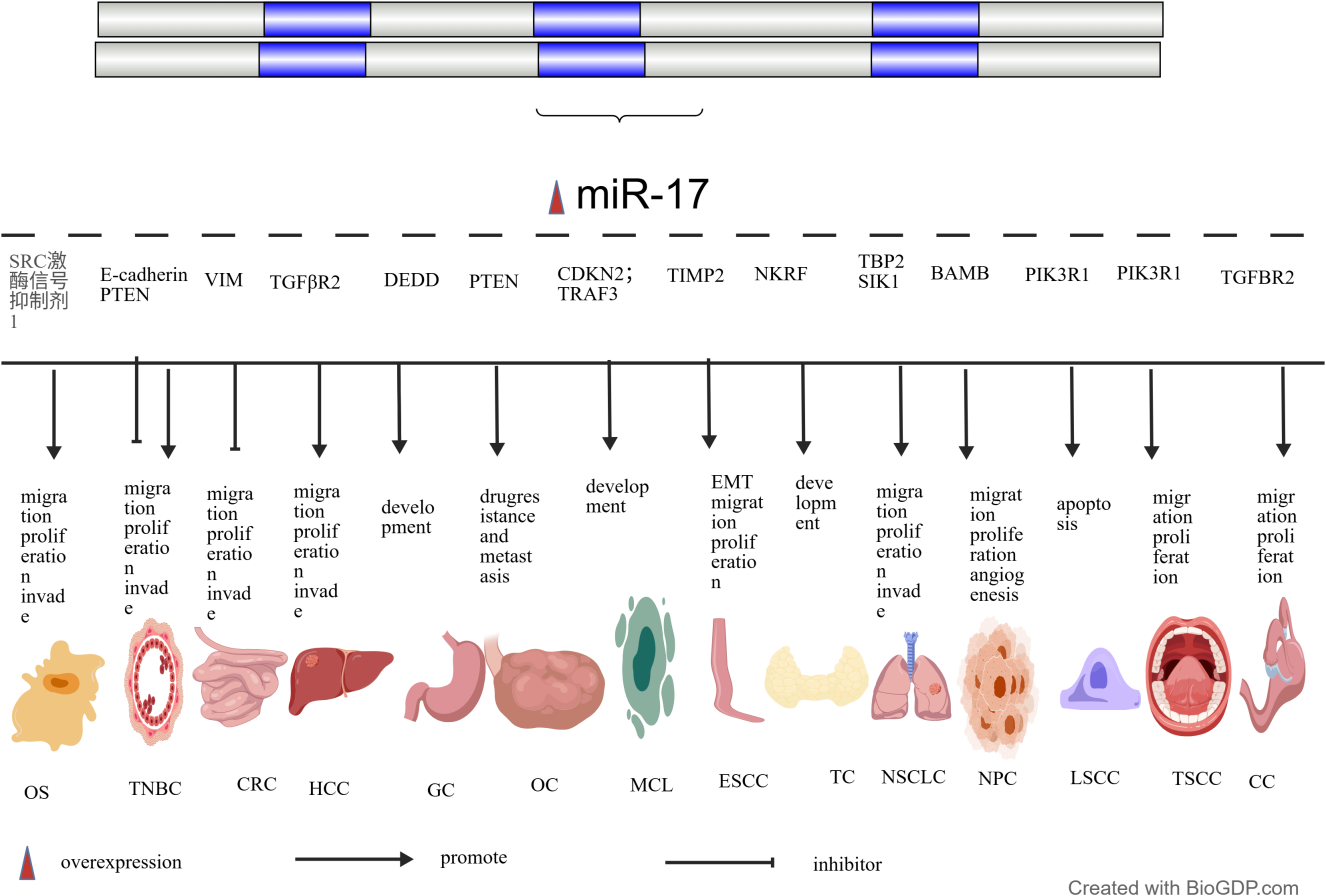
Biological effects and molecular targets of miR-17 across various cancer types; NPC, Nasopharyngeal carcinoma; LSCC, Laryngeal squamous cell carcinoma;.

As a pivotal molecule within the ceRNA network, miR-17 not only participates in the regulation of cancer-related processes via the ceRNA axis through its “molecular sponge” mechanism, but its dysregulation is also closely associated with the initiation and progression of multiple cancers (see **Table 2**, **Figure 2**). Specifically, miR-17 exerts a decisive influence on core biological processes, including cell proliferation, apoptosis, migration, invasion, metastasis, and cell cycle progression by regulating the expression of downstream target genes. This provides direct evidence for its molecular mechanism in shaping cancer biological characteristics (40, 42).

**Table 2:** miR-17 target genes and their mechanisms in different cancer types.

First author/year	Cancer type	miR-17 target gene	miR-17 mechanism	(Refs.)
Zhao, et al.2019	OS	SKI-1	Targets and inhibits the SRC kinase signal pathway. Inhibits cell proliferation and EMT.	(40)
Zhong, et al.2017	BC	E-cadherin	Targets E-cadherin inhibition, counteracting doxorubicin's suppression of EMT	(41)
Bao, et al.2021	TNBC	PTEN	Targets PTEN inhibition, activates PTEN/Akt signalling axis, promotes EMT and cell migration/invasion.	(42)
Jin,et al.2020	CRC	VIM	Directly binds VIM mRNA 3'UTR, downregulates its expression, inhibits EMT and cell migration/invasion.	(43)
Liu,et al.2023	HCC	TGFβR2	Negatively regulates TGFβR2 expression, inhibiting cell proliferation, migration, invasion, and EMT while promoting apoptosis and slowing tumour progression.	(44)
Wu,et al.2018	GC	DEDD	Promoting gastric cancer progression through targeted DEDD	(45)
Ying,et al.2015	OC	PTEN	By downregulating PTEN and activating the AKT signaling pathway, it mediates drug resistance and metastatic ability.	(46)
Wang,et al.2021	ESCC	TIMP2	Overexpression of miR-17 promotes ESCC cell proliferation, invasion, and migration, and epithelial-mesenchymal transition.	(47)
Yan, et al.2015	TCr	NKRF	Reshaping the transfer mechanism of pulmonary pro-inflammatory microenvironment by activating NF-KB signaling	(48)
Ye, et al.2020	lung cancer	TBP2	Believe the anticancer effect of TBP2 through negative regulation and promotes the progression of lung cancer.	(49)
chen ,et al.2025	NSCLC	SIK1	Directly binds to SIK1 mRNA 3'UTR to inhibit its mRNA and protein expression; promotes cell proliferation, migration, and invasion, and inhibits cell apoptosis.	(50)
Duan ,et al.2019	NPC	BAMB	Directly binds to the 3'UTR of BAMBI mRNA to inhibit BAMBI protein expression; activates AKT/VEGF-signaling, promotes angiogenesis, cell proliferation, and migration.	(51)
Wang ,et al.2020	Lscc	PIK3R1	By restoring PIK3R1 expression, blocking the PI3K/AKT pathway, and inducing cell apoptosis.	(52)
Pang ,et al.2020	Sct	Beclin-1	By inhibiting the key autophagy-initiating molecule Beclin-1, autophagy-dependent cell death under hypoxic conditions is weakened, while promoting proliferation and migration.	(53)
Cai ,et al.2018	CC	TGFBR2	By targeting and silencing TGFBR2, blocking the anti-cancer signal of TGFβ, the proliferation and migration potential of CC cells can be released.	(54)

**Figure 3:**
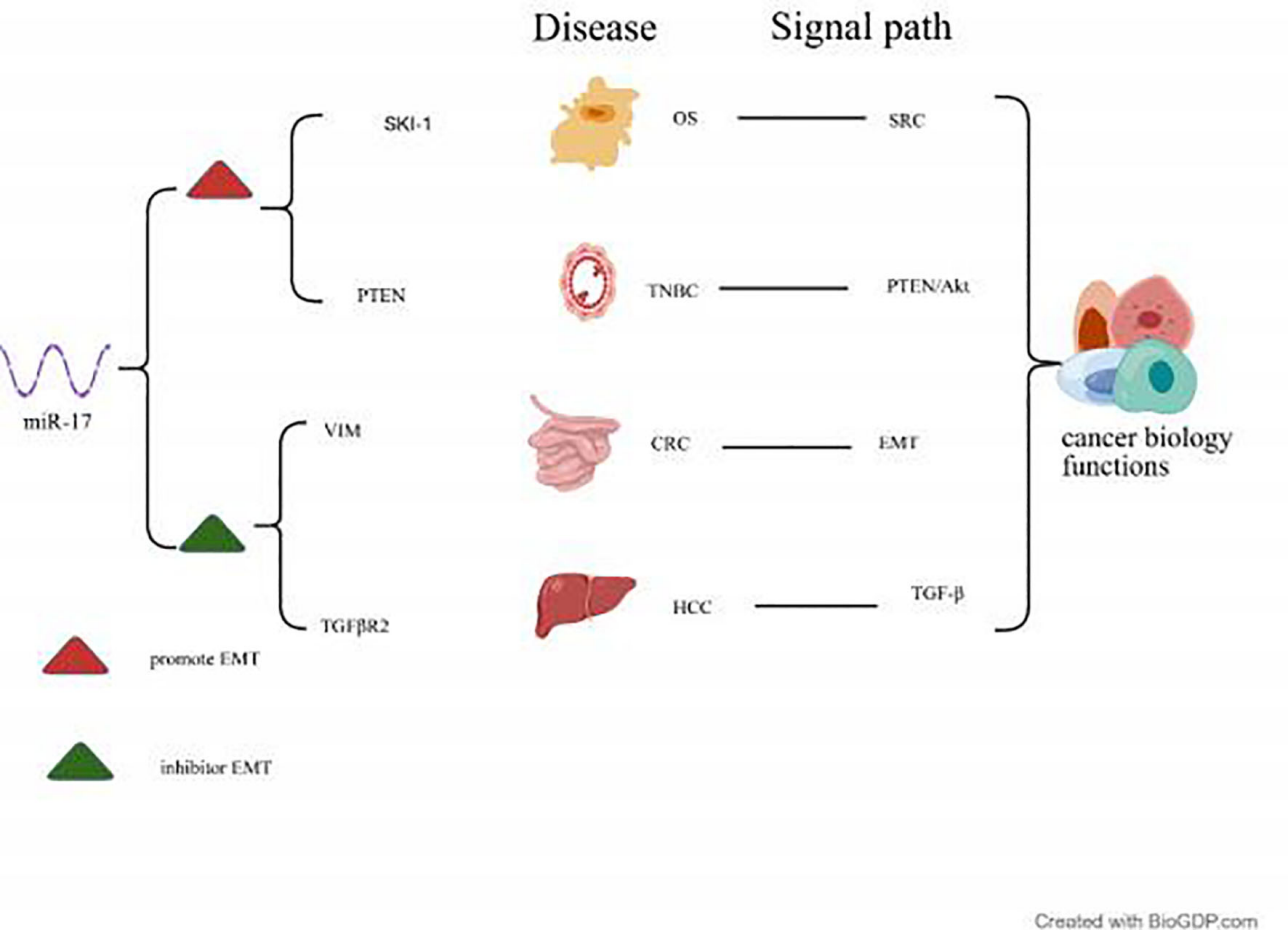
The involvement of miR-17 in the cancer EMT process.

miR-17 extensively participates in the EMT process by targeting diverse signalling pathways, bidirectionally regulating tumour cell invasion, migration, and stem cell-like properties. On the one hand, to promote EMT effects, for instance, Zhao et al. discovered that miR-17 promotes proliferation and EMT in human osteosarcoma (OS) cells by targeting Ski-like oncogene 1(SKI-1). Similarly, research by Bao et al. (42) on triple-negative Breast Cancer (TNBC) demonstrated that miR-17 targets and silences the tumour suppressor gene PTEN, thereby activating the PTEN/Akt signalling axis and significantly promoting EMT alongside tumour cell migration and invasion. Furthermore, Zhong et al. (41) demonstrated that miR-17 promotes EMT via the PTEN/Akt signalling axis, thereby enhancing the migration and invasive capabilities of TNBC cells. On the other hand, to suppress EMT activity, Jin et al. (43) discovered in CRC that miR-17 directly binds to the 3’ UTR of vascular endothelial growth factor receptor (VEGFR) mRNA and downregulates its expression, thereby inhibiting EMT and cell migration/invasion. Furthermore, *in vivo* experiments demonstrated a reduction in tumour metastatic potential to the liver. Liu et al. (44) discovered that miR-17 negatively regulates Transforming Growth Factor Beta Receptor 2 (TGFβR2) expression, thereby blocking the oncogenic branch of the Transforming Growth Factor Beta (TGF-β) signalling pathway. This ultimately inhibits the proliferation, migration, invasion, and EMT of HCC cells, promotes apoptosis, and slows tumour progression. See **Figure 3** for details.

In addition to EMT, miR-17 also participates in the malignant progression of various cancer types by targeting specific genes. For instance, Cai et al. (54) demonstrated that miR-17, by targeting and silencing TGFBR2, disrupts the tumour-suppressing signalling of TGF-β, thereby unleashing the proliferative and migratory potential of cervical cancer (CC) cells. Concurrently, Ye et al. (49) discovered that miR-17 negatively regulates the tumour suppressor gene latent transforming growth factor beta-binding protein 2 (TBP2), thereby relieving its growth-inhibitory effect on LC cells and directly promoting tumour progression. Chen et al. (50) demonstrated in their study of NSCLC that miR-17 directly binds to the 3’UTR of salt-induced kinase 1 (SIK1) mRNA, thereby suppressing its expression. This promotes cellular proliferation, migration, and invasion while simultaneously inhibiting apoptosis. Furthermore, Wu et al. (45) discovered that miR-17 promotes GC progression by targeting death effector domain protein (DEDD). Ying et al. (46) demonstrated that miR-17 downregulates PTEN to activate the AKT pathway, thereby mediating tumour drug resistance and metastasis in ovarian cancer (OC). Concurrently, Duan et al. (51) observed that miR-17 targets bone morphogenetic protein and activin membrane-binding inhibitor bone morphogenetic protein and activin membrane-binding inhibitor (BAMBI), thereby activating the AKT/vascular endothelial growth factor-A(VEGF-A) signalling pathway to promote angiogenesis, cellular proliferation, and migration.

In summary, miR-17 exhibits functional heterogeneity in cancer progression, acting both as an oncogene and a tumour suppressor through its involvement in the ceRNA network and regulation of target genes. The diversity of its molecular mechanisms provides potential targets for precision cancer therapy. Future research should further elucidate the specific regulatory networks of miR-17 across different cancer types, thereby laying the groundwork for developing miR-17-based targeted therapeutic strategies.

## 5 The relationship between miR-17 and MT

### 5.1 Expression in different M T

MiR-17 exhibits widespread dysregulation across multiple human malignancies, with its expression levels being closely associated with tumour initiation, progression, and patient prognosis. Quantitative studies indicate that miR-17 is associated with NSCLC (55), GC (56), bladder cancer (bladder Ca) (57), BC (58), and so on. Significantly up-regulated in multiple solid tumour tissues, including. This state of high expression is frequently associated with more aggressive clinical pathological characteristics: in prostate cancer (PCa), it correlates positively with higher Gleason scores (59); In Ca, it is associated with higher tumour stage andpathological grade (56);In GC, it is significantly correlated with primary tumour diameter, T stage, lymph node metastasis, and TNM staging (60). Moreover, its expression levels carry distinct prognostic significance: elevated expression in the serum of NSCLC patients correlates with lymph node metastasis, and patients with metastasis exhibit a markedly reduced five-year survival rate (61). High expression of NPC (62) is also associated with lower overall survival rates.

However, the expression pattern of miR-17 is not consistent across all cancer types, demonstrating significant tumour heterogeneity. Within CRC, research conclusions remain divided: some studies indicate its expression is upregulated in cancerous tissue, promoting cell proliferation and invasion (63); other studies, however, have found its expression to be lower than in adjacent non-cancerous tissue, with low expression associated with deeper invasion, more advanced TNM staging, and lymph node metastasis (64). A similar contradiction has been observed in HCC: most studies report that miR-17 expression is upregulated (65); however, studies have also indicated that miR-17 is downregulated in HCC, with low expression levels significantly associated with higher Edmondson grades, microvascular invasion, and poor prognosis (66). miR-17 exhibits a trend of high expression in the vast majority of MT and is closely associated with tumour progression, metastasis, and poor prognosis. Differences in expression are observed in certain cancers, such as CRC and HCC, potentially attributable to tumour heterogeneity, distinct molecular profiles, and variations in research methodologies.

### 5.2 Mechanisms of expression regulation

#### 5.2.1 Regulation of transcriptional levels

Multiple transcription factors can regulate miR-17. Among these, the primary transcription activator is c-Myc. The expression of c-Myc is frequently dysregulated in tumours, promoting proliferation and inhibiting terminal differentiation. The transcription factor c-Myc is a central regulator of cell proliferation, and its expression is abnormally upregulated in numerous tumours. c-Myc exerts its transcriptional function by binding to the E-Box sequence within the promoter region of target genes, serving as a key transcriptional activator for miR-17 (67). In PC, BC, B-cell lymphoma(BCL), and chronic myeloid leukaemia(CML), the MYC proto-oncogene, bHLH transcription factor(c-Myc) suppresses downstream target genes (such as the tumour suppressor gene Glypican-5(GPC5) by activating this cluster of expression, thereby sustaining the tumour’s survival and proliferative state (22, 68, 69).

#### 5.2.2 Epigenetic regulation

The expression of miR-17 is subject to precise regulation by epigenetic mechanisms, with DNA methylation being a key factor influencing its expression. The hypomethylated state of the promoter region is typically associated with the activation and upregulation of gene expression (70). Moreover, lncRNAs and circRNAs may indirectly regulate miR-17 activity through competitive ceRNA mechanisms, constituting another layer of complex epigenetic regulatory networks (71). Hypermethylation of specific genes such as Vimentin(VIM)and Septin 9 (SEPT9), or hypomethylation of the Long Interspersed Nuclear Element 1( LINE1 ) genomic repetitive sequence, both influence the expression of miR-17 (72).

#### 5.2.3 Post-transcriptional regulation

At the post-transcriptional level, RNA-binding proteins exert fine-tuned regulation over miR-17’s function by influencing the stability of miRNA precursors, their processing pathways, or their interactions with target mRNAs.HuR is an important RNA-binding protein that regulates gene expression by binding to and stabilising its target RNA. HuR can directly bind to and stabilise the precursor transcript of miR-17 (73). Moreover, in neuroblastoma, HuD (ELAVL4) competitively binds to the 3’ UTR of the oncogene Neuroblastoma Myc(N-Myc) mRNA with miR-17, establishing an antagonistic regulatory relationship: HuD stabilises N-Myc mRNA (positive regulation), while miR-17 promotes its degradation (negative regulation), jointly exerting fine-tuned control over N-Myc expression levels (74).

## 6 The core function of miR-17 in tumour development

### 6.1 Driving cell proliferation and inhibiting apoptosis

The core mechanism by which miR-17 drives MT progression lies in its coordinated targeting and suppression of multiple key tumour suppressor genes controlling cell cycle and survival signalling pathways, thereby establishing a potent oncogenic regulatory network. Cell cycle disruption is a key feature of tumourigenesis, miR-17 directly influences cell cycle progression by interfering with cyclin-dependent kinase (CDK) cyclin-CDK complexes and associated signalling pathways.p21 and p27 are required CDK inhibitors.miR-17 promotes cell growth in haematological malignancies such as CML and BCL (75), as well as in solid tumours including nasopharyngeal carcinoma (76) and OS (77), By inhibiting p21, miR-17 may also simultaneously suppress p21 and TP53-induced Nuclear Protein 1(TP53INP1), thereby synergistically promoting GC cell proliferation and inhibiting apoptosis (78). Retinoblastoma 1(RB1)is another core protein regulating G1 progression and a classic tumour suppressor. The RB1 tumour suppressor negatively regulates the cell cycle and is inactivated in numerous human tumours. Overexpressed miR-17 directly binds to the 3’UTR of RB1 mRNA, thereby reducing RB1 translation and consequently diminishing RB1 protein expression (79). Liu et al. discovered that (80) miR-17 expression positively correlates with SRY-Box Transcription Factor 4(SOX4) expression in PC patients, whilst negatively correlating with RB1 expression. Mechanistically, SOX4 transcribes to upregulate miR-17 expression within PC cells, subsequently directly downregulating RB1 protein. This establishes a functional axis of ‘SOX4 → miR-17 → RB1’, thereby promoting the proliferation, migration, and invasion of PC cells.

miR-17 further modulates cell cycle progression via core driver proteins and CDKs. In BC cells, the cyclin D/cyclin-dependent kinase 4 and 6 (CDK4/6) and retinoblastoma (RB) pathways play a crucial role in the proliferation of normal breast tissue, particularly in epithelial cell growth. Asberjer et al. (81) discovered that downregulating miR-17 inhibits CDK4/6 activity, hereby suppressing the proliferation of BC cells and inducing G1 cell cycle arrest. Concurrently, research by Yu et al. (82) demonstrated that miR-17 inhibits BC cell proliferation by suppressing the translation of cyclin D1. Furthermore, Wang et al. (83) discovered that knockdown of the LncRNA NHG16 suppressed cyclin D1 expression by sponging miR-17 in a small-interference RNA(SiRNA)- dependent manner, thereby inhibiting the development of oralsquamous cell carcinoma (OSCC)both *in vitro* and *in vivo*. Similarly, research by Huan et al. (84) confirmed that miR-17 negatively regulates the cell cycle in head and neck squamous cell carcinoma (HNSCC) by directly targeting Cyclin G2(CCNG2), thereby increasing the proportion of cells in the G2/M phase and reducing the proportion in the S phase. Hu et al. (85) discovered that miR-17 expression was elevated in endometrial tissue from patients with adenomyosis, potentially influencing apoptosis and cyclin expression by targeting PTEN regulation.

miR-17 also influences the cell cycle through key signalling pathways such as Wnt/β-catenin and PI3K/AKT. For instance, Sun et al. (86) observed that increasing downregulation of miR-17, accompanied by elevated Phosphorylated Akt (P-Akt) and Akt expression, enhanced the survival and migration rates of glioblastoma (GBM) cells. Concurrently, research by Mu et al. (87) demonstrates that the Wnt/β-catenin signalling pathway is crucial for regulating cell proliferation and differentiation. By upregulating miR-17, it mediates the Wnt/β-catenin pathway to exert a proliferative effect on cells. Furthermore, Yu et al. (88) demonstrated that miR-17-activated Wnt/β-catenin pathways promote the progression of liver fibrosis. Yuan et al. (89) discovered that miR-17, upregulated via the LncRNA miR-17HG, promotes CRC cancer progression by activating the Wnt/β-catenin signalling pathway. Similarly, Chen et al. (90) discovered that miR-17 downregulation inactivates Wnt/β-catenin signalling by targeting kinesin-like motor protein family member 23 (KIF23), thereby alleviating apoptosis and fibrosis induced by high glucose in human mesangial cells.

## 7 miR-17 and signal pathways

**Figure 4:**
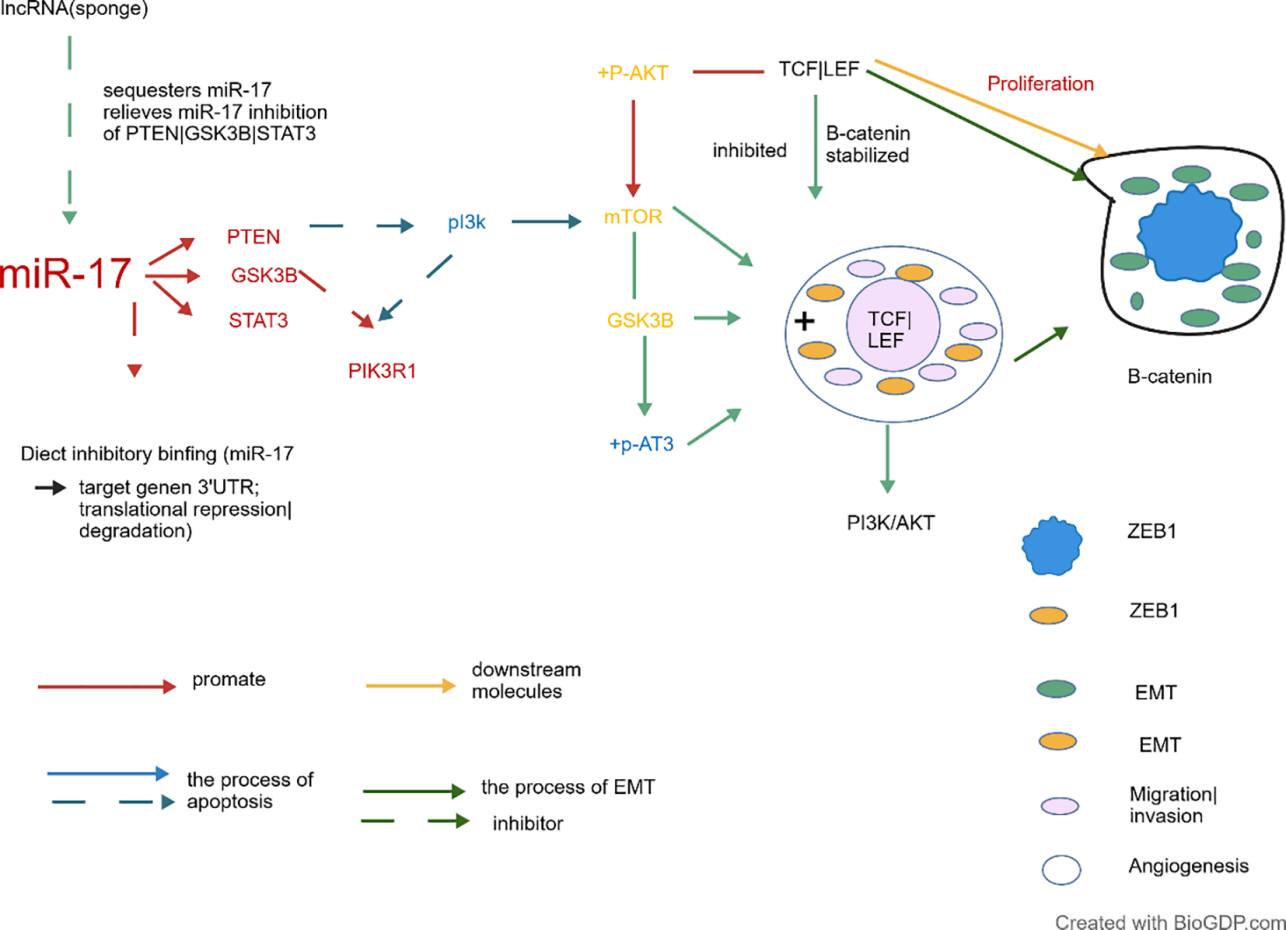
The downstream signalling pathway is regulated by miR-17.

Cellular signalling constitutes the core mechanism through which cells respond to internal and external stimuli, indirectly influencing gene expression by regulating transcription factor activity. miRNAs, as pivotal regulatory molecules, play a significant role within the signalling pathway networks of both normal and cancerous cells. The following outlines the regulatory mechanisms of miR-17 on cellular signalling pathways (For details, see **Figure 4**.).

MiR-17 activates the PI3K/AKT pathway by targeting PTEN and plays a pro-survival/invasive role in different cancers: Zhang et al. (91) and Lu et al. (92) confirmed miR-17’s pro-survival effect in PC via the PI3K/AKT pathway. The pro-survival function of miR-17 is integrated into a complex ncRNA regulatory network. Zhang et al. (93) discovered that the lncRNA HORAIRM1 acts as a ceRNA for miR-17, thereby blocking the PI3K/AKT pathway by relieving its inhibition on PTEN, thus suppressing GC progression. Concurrently, Lu et al. (94) discovered that the lncRNA NEAT1 promotes gastric cancer cell survival and migration by binding to miR-17, thereby reversing the activation of the PI3K/AKT and GSK3β pathways. Furthermore, Wang et al. (95) discovered that the PTEN pseudogene PTENP1 suppresses the PI3K/AKT pathway and induces apoptosis and autophagy in HCC cells by sequestering miR-17 and restoring PTEN expression.

**Figure 5:**
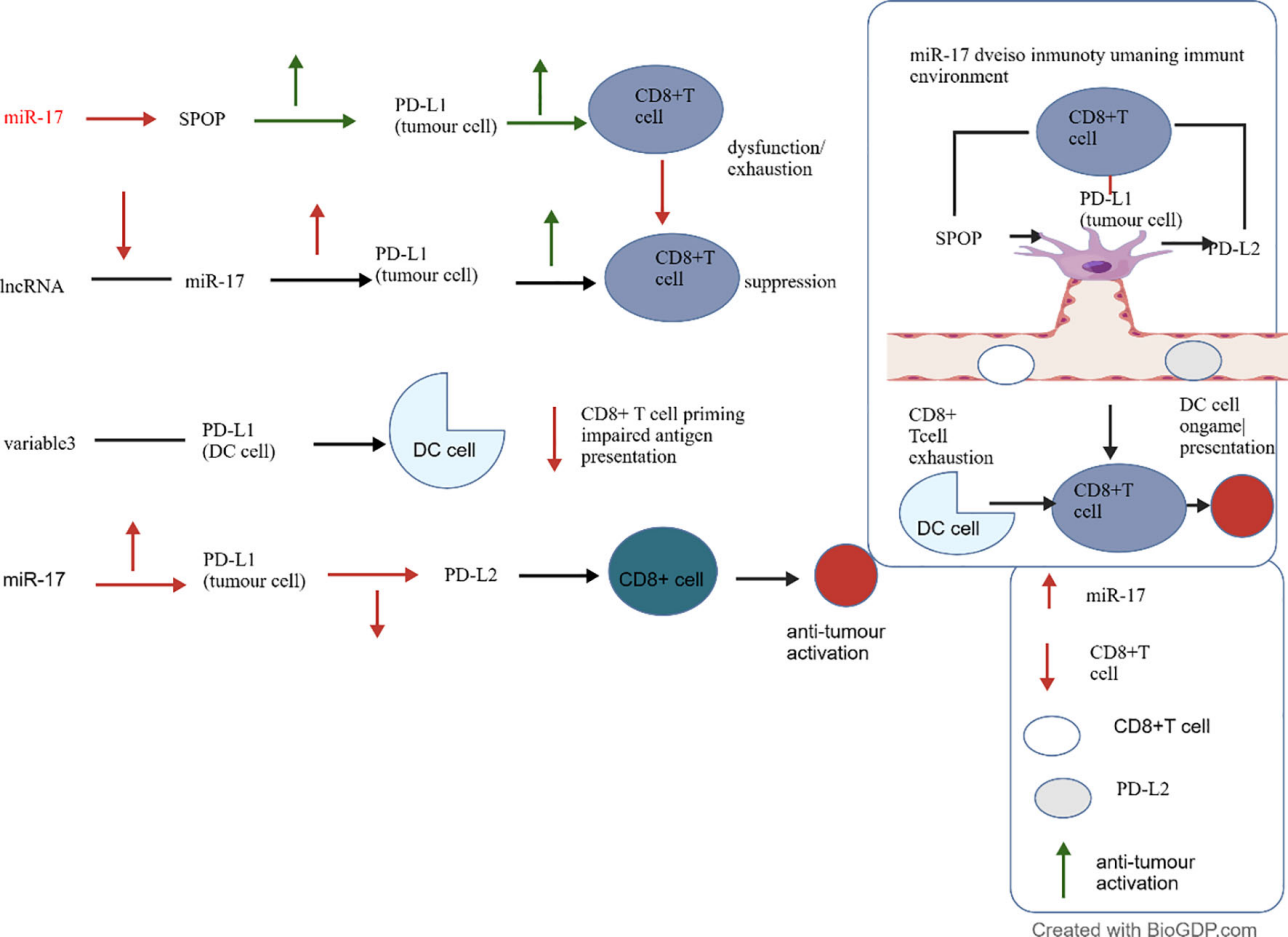
Regulatory Network of miR-17 in the Tumour Microenvironment.

In addition to PTEN, miR-17 may also target other key molecules within the PI3K/AKT pathway and influence drug efficacy. For example, Zhang et al. (96) discovered that miR-17 inhibits the proliferation of LSCC and promotes apoptosis by directly targeting PIK3R1 (a regulatory subunit of PI3K). Furthermore, Ren et al. (97) discovered that upregulating miR-17 attenuates metformin’s inhibitory effect on invasion in HCC by activating the PTEN/PI3K/Akt pathway. miR-17 also regulates the proliferation, apoptosis, and drug resistance of PC, bladder Ca, NSCLC, and Pca cells by inhibiting the JAK-STAT3 pathway or inducing Signal Transducer and Activator of Transcription 3(STAT3) degradation (98–100). In summary, miR-17 regulates the PI3K/AKT and JAK-STAT3 pathways, with LncRNA and pseudogenes participating in its function, providing potential targets for cancer therapy. The regulatory network of miR-17 in the tumour microenvironment is illustrated in **Figure 5**.

## 8 Targeted apoptosis proteins and factors

MiR-17 plays a pivotal role in regulating apoptosis and cell survival. Its function is mediated by targeting the Bcl-2 protein, the apoptotic effector proteins Caspase-3 and Caspase-9, and the upstream pro-apoptotic factor Bim Its action exhibits a high degree of environmental and tissue specificity: In most scenarios, it exerts anti-apoptotic effects by either suppressing apoptosis through promoting Bax and Caspase-3/9 expression whilst downregulating Bcl-2 levels, or by diminishing apoptosis rates via targeted inhibition of Toll-like receptor 4(TLR4) to attenuate the nuclear factor kappa-B( NF-κB) signalling pathway (101). Alternatively, exosome-mediated uptake of circZNRF1 may regulate Bcl-2 binding to exert anti-apoptotic effects (102).It can also inhibit the survival factor insulin-like growth factor 1(IGF-1) from upregulating Bcl-2 and downregulating Caspase-3 (103), or affect the expression of PTEN and apoptosis-related proteins to promote cell proliferation (104). Under specific conditions, it exhibits pro-apoptotic effects, such as inhibiting Bcl-2 expression and activating Caspase-3 cleavage (105)or downregulating Bcl-2 and STAT3 expression to enhance apoptosis (98).It may also synergise with miR- 16 to induce apoptosis via the Caspase-3 pathway (106). Furthermore, miR-17 enhances cellular viability by directly suppressing Bim (107) whilst oncogene-driven miR-17 inhibits Bim to shield cells from apoptosis (108).This bidirectional regulation underscores its pivotal role as a central regulatory hub (80).Bidirectional regulation within complex networks and disease-specific functional clusters is deeply integrated into broader signalling networks, potentially yielding bidirectional or even contradictory outcomes depending on cell type and microenvironmental signalling. .

## 9 miR-17 and chemotherapy/radiotherapy resistance in cancer

Despite ongoing advances in cancer treatment technologies, chemotherapy remains the cornerstone therapy for advanced cancers. However, chemotherapy resistance continues to be one of the key obstacles limiting treatment efficacy (109, 110). Recent studies indicate that miRNAs play an increasingly significant role in regulating cancer cells’ therapeutic response, particularly in the mechanisms of chemotherapy resistance. By targeting and modulating the expression of relevant genes, miRNAs directly influence tumour cells’ sensitivity to chemotherapeutic agents (111). Its core functional mechanism operates as follows: miR-17, acting as a post- transcriptional regulator of gene expression, can bind to the 3’-UTR region of target genes. This binding inhibits mRNA translation or promotes mRNA degradation, thereby modulating key cellular pathways such as apoptosis, proliferation, and DNA damage repair. Ultimately, this mediates chemoresistant or chemosensitive phenotypes (112).

MiR-17 plays a complex role in regulating chemotherapy sensitivity and resistance across multiple cancer types. For instance, miR-17 mediates resistance to chemotherapeutic agents (such as cisplatin) and targeted therapies in various tumours by regulating multiple key molecules involved in apoptosis and survival, including Bim and PTEN (113). Concurrently, Weng et al. (114) discovered that the traditional Chinese medicine compound oridonin can induce apoptosis by inhibiting miR-17 and reverse chemoresistance by de-suppressing Bcl-2 Interacting Mediator of Cell Death Short Form (Bim-S). Furthermore, research by Shuang et al. (115) confirmed that miR-17 suppresses Bim expression by directly binding to the Bim 3′-UTR, thereby reducing the sensitivity of human OC SKOV3-TR30 cells to paclitaxel. Similarly, Dai et al. (116) discovered that MYC/miR-17 form a positive feedback loop to maintain low Bim expression, thereby permitting cell proliferation. STAT3 and miR-17 form a positive feedback loop: STAT3 upregulates miR-17, which in turn upregulates STAT3, collectively mediating tumour cell resistance to Mitogen-Activated Protein Kinase Kinase(MEK)inhibitors.”miR-17 may also regulate other drug resistance pathways. For instance, Wan et al. (117) observed that interference with miR- 17 potentially aids in overcoming glucocorticoid resistance in B-cell precursor acute lymphoblastic leukaemia (BCP-ALL). Concurrently, research by Jiang et al. (118) demonstrated that the miR-17 family regulates cisplatin resistance and metastasis in NSCLC by targeting TGF-β receptors.

The role of miR-17 in cancer chemotherapy and radiotherapy highlights its complex regulatory functions, which are influenced by tumour type, therapeutic agents, and cancer cell-specific characteristics. Specifically, the expression of miR-17 across different cancer types may be influenced by multiple factors, including the drug resistance characteristics of tumour cells and alterations within the tumour microenvironment. Therefore, the mechanism of action of miR-17 as a potential therapeutic target warrants further investigation. In summary, miR-17’s dual role in chemotherapy and radiotherapy underscores its potential as a therapeutic target, with implications for overcoming drug resistance and guiding personalized treatment strategies. An in-depth investigation into the mechanisms of miR-17 and its interactions with chemotherapeutic agents and radiotherapy will provide valuable insights for overcoming drug resistance in cancer treatment and offer new directions for future therapeutic strategies.

## 10 Clinical translation prospects for targeting miR-17

### 10.1 As a diagnostic and prognostic biomarker

Detection of miR-17 in patient serum, plasma, or exosomes demonstrates its potential as a non-invasive liquid biopsy biomarker for early cancer diagnosis and prognostic assessment. Studies by Li et al. (119) revealed that miR-17 exhibits elevated expression in serum samples from both GC patients and those with intestinal metaplasia, compared to healthy controls. Consequently, serum miR-17 demonstrates discriminatory power between GC and intestinal metaplasia patients versus healthy controls, suggesting that the miR-17 cluster holds potential as a serum biomarker for the early detection of GC.

Similarly, miR-17 is significantly elevated in the plasma of CRC patients and may serve as a biomarker for CRC screening (120). Research has confirmed that plasma levels of miR-17 are significantly elevated in patients with Pa, ESCC, and GC compared to control volunteers. Furthermore, in these three cancer types, plasma concentrations of miR-17 in post-operative samples were markedly lower than those in pre-operative samples (121–123). This confirms that miR-17 can serve as a biomarker for the screening and monitoring of MT.

### 10.2 As a therapeutic target

#### 10.2.1 Antisense oligonucleotides

The ASO inhibitor of miR-17 has demonstrated antitumour effects in clinical models. For example, Wan et al. (124) found that ASO may inhibit proliferation and induce apoptosis in lung cancer cells by suppressing the oncogenic miR-17. Furthermore, Han et al. (125) discovered that circ LONP2, formed from exons 11–12 of LONP2, enhances the *in vitro* invasive capacity of cancer cells, with its core function dependent on regulation via the miR-17 pathway. Targeting circLONP2 through anti-AS inhibition significantly reduces the *in vitro* invasive and metastatic capabilities of CRC cells. Concurrently, Locked Nucleic Acid (LNA) gapme RASOs induce ribonuclease H-mediated degradation of the MIR17 Host Gene (MIR17HG) primary transcript, thereby blocking the biosynthesis of the miR-17and inhibiting CRC tumour growth (126).10.2.2 Targeted miRNA processing and functional complexes.

Developing small-molecule drugs to disrupt the binding of miRNA to Argonaute (AGO) proteins or interfere with their biogenesis represents another significant avenue of research. Among humans, AGO2 is considered the sole cleaver, whilst AGO3 is scarcely capable of cleaving RNA (127). Argonaute2 protein is crucial for embryonic development, stem cell maintenance, and cellular differentiation. Under stable and prolonged overexpression, AGO2 induces the production of multiple miRNAs in 7T cells, such as those within the let-7 family.

On the other hand, the expression of numerous miRNAs remains insensitive to elevated Argonaute levels, with miR-17 even being downregulated. This downregulation may result from let-7-mediated suppression of Myc expression, as Myc actively regulates the transcription of miRNA clusters (128). Growth Factor Receptor-Bound Protein 2(GRB2) enhances miR-17 expression. GRB2 forms a direct interaction with AGO2, mediated by the Src Homology 3 domain (SH3). Complex formation is entirely dependent on GRB2 concentration. Therefore, strict regulation of GRB2 expression is essential to eliminate abnormal oncogene expression and cellular proliferation (129). Research by Iosue et al. revealed that silencing AGO2 impairs the function of miR-17 and promotes cellular differentiation (130). These studies indicate that targeting AGO2 or its regulatory factors can indirectly modulate the miR-17 functional network.

#### 10.2.3 Combined treatment strategy

Combining miR-17 inhibitors with standard chemotherapy, radiotherapy, or immune checkpoint inhibitors holds promise for overcoming resistance and enhancing therapeutic efficacy. For instance, Sun et al. (131) discovered that the miR-17 targeting METTL14 (methyl transferase-like protein 14 in colorectal cancer)/miR-17/MFN2 (mitochondrial fusion protein 2) signalling axis can restore the chemotherapeutic sensitivity of CRC cells to 5-FU. Concurrently, Weng et al. (114) observed that elevated miR-17 expression diminishes cellular sensitivity to etoposide; downregulating miR-17 expression via miRNA inhibitors or oridone restores chemotherapy sensitivity to etoposide. Similarly, research by Dai et al. (116) revealed that STAT3-regulated miR-17 serves as a key driver of resistance to MEK inhibitors such as AZD6244. Inhibiting miR-17 reverses the sensitivity of resistant cells to AZD6244 by inducing Bim expression and Poly (ADP-Ribose) Polymerase (PARP) cleavage. Concurrently, Yin et al. (132) discovered that let-7 and miR-17 respectively influence self- renewal and gefitinib resistance by regulating Myc and Cyclin-Dependent Kinase Inhibitor 1A(CDKN1A). On the one hand, low let-7 levels promote Myc expression to aid in maintaining an undifferentiated state. On the other hand, elevated miR-17 levels reduce CDKN1A expression, thereby contributing to the preservation of proliferative potential. Consequently, the combined effects of low let-7 and high miR-17 regulate self-renewal by promoting cancer stem cell expansion, thereby conferring protection against gefitinib-induced cytotoxicity and generating gefitinib resistance.

## 11 Future and outlook

Research into miR-17 across various tumours represents a dynamic field with multifaceted potential for future studies and clinical applications. miR-17 and its specific miRNAs offer promising therapeutic targets for developing novel treatments for diverse cancers. The expression levels of miR-17 may serve as diagnostic and prognostic biomarkers for various cancers. Continued investigation into its clinical significance and validation within large patient cohorts is of paramount importance. Understanding the mechanisms by which miR-17 promotes metastasis and the role of EMT in various tumours facilitates the development of therapies targeting these processes, thereby improving patient outcomes. Exploring potential synergies between miR-17-targeted therapies and existing cancer treatments, such as chemotherapy or immunotherapy, holds promise for enhancing the efficacy of current therapeutic approaches. Tailoring treatment strategies to a patient’s specific miRNA expression profile may yield more personalised and effective therapeutic approaches. Regarding drug resistance, further research into miR-17 and its impact on drug response, alongside the development of personalised therapies based on patients’ miRNA profiles, could enable treatments that modulate miR-17 expression or activity. This approach holds promise for overcoming chemotherapy resistance and enhancing therapeutic outcomes across diverse patient populations.

miR-17 drives malignant progression in tumours through a complex network of synergistic interactions at multiple levels. Future research should delve deeper into the distinct and overlapping functions of miR-17 within specific tumour contexts, while developing targeting strategies with enhanced selectivity and reduced toxicity. Concurrently, elucidating its precise role in immune regulation within the tumour microenvironment will open new avenues for immunotherapy combination approaches. Further investigation into how miR-17 promotes chemotherapy resistance and its impact on mitochondrial homeostasis may reveal novel therapeutic targets. A deeper understanding of miR-17 as a “signalling hub” will undoubtedly advance the era of RNA-based precision cancer diagnosis and treatment.

## 12 Summary

As a prototypical oncogenic miRNA, miR-17 plays a central driving role in malignant tumours such as lymphoma, lung cancer, CRC, and BC. It inhibits apoptosis and promotes G1/S transition to drive unlimited proliferation of tumour cells by targeting tumour suppressor genes and cell cycle regulators.

Concurrently, it targets molecules associated with EMT and activates key signalling pathways, including PI3K/AKT, Wnt/β-catenin pathways, to enhance tumour cell migration and invasive capacity. It may also participate in ncRNA cross-regulatory networks, being adsorbed by LncRNAs and circRNAs as molecular sponges or regulating their stability and biogenesis. This further amplifies OE and induces tumour cell resistance to chemotherapy and radiotherapy, ultimately synergistically promoting tumour angiogenesis and microenvironmental remodelling. In clinical applications, miR-17 demonstrates potential for early cancer diagnosis, particularly serving as a non-invasive biomarker in GC and lung cancers (56). When targeted therapeutically, inhibiting miR-17 expression enhances the efficacy of chemotherapy or targeted therapies while improving patient prognosis (133). However, its clinical application remains challenged by factors such as incomplete elucidation of its mechanisms of action. Future research must further unravel its molecular regulatory networks to advance precision diagnostics and therapeutic translation. In recent years, an increasing number of studies have further revealed the regulatory roles and clinical application potential of miR‑17 in different tumors, which provide a theoretical basis for targeted therapy and biomarker research (134–146).
