# Correction: miR-17: from developmental regulatory hub to molecular engine driving tumourigenesis

**DOI:** 10.3389/fonc.2026.1889109

**Published:** 2026-06-15

**Authors:** Min Zhang, Yurong Ma, XuanYu Chen, Tiansi Sun, XiYi Wang, Feng Hui Wang

**Affiliations:** Yan’an Medical College of Yan’an University, Yan’an, Shaanxi, China

**Keywords:** diagnostic markers, miR-17, signalling pathways, target genes, tumor biology

In the published article, there was an error in the affiliation(s). All authors were incorrectly listed with multiple affiliations (#1, #2, #3). Instead of “#1, #2, #3”, it should be “¹ Yan’an Medical College of Yan’an University, Yan’an, Shaanxi, China”. The affiliations #2 and #3 are removed for all authors. The authors apologize for this error and state that this does not change the scientific conclusions of the article in any way.

The original article has been updated.

In the published article, there was an error regarding the author roles: Min Zhang is the first author and was erroneously marked as a corresponding author. Feng Hui Wang¹* is the correct corresponding authors. The corrected author list is: “Min Zhang¹, Yurong Ma¹, XuanYu Chen¹, Tiansi Sun¹, XinYi Wang¹, Feng Hui Wang¹*” The authors apologize for this error and state that this does not change the scientific conclusions of the article in any way.

The original article has been updated.

